# Accuracy of real-time PCR, Gram stain and culture for *Streptococcus pneumoniae, Neisseria meningitidis* and *Haemophilus influenzae* meningitis diagnosis

**DOI:** 10.1186/1471-2334-13-26

**Published:** 2013-01-22

**Authors:** Henry M Wu, Soraia M Cordeiro, Brian H Harcourt, MariadaGloriaS Carvalho, Jailton Azevedo, Tainara Q Oliveira, Mariela C Leite, Katia Salgado, Mitermayer G Reis, Brian D Plikaytis, Thomas A Clark, Leonard W Mayer, Albert I Ko, Stacey W Martin, Joice N Reis

**Affiliations:** 1 Division of Bacterial Diseases, National Center for Immunization and Respiratory Diseases, CDC, 1600 Clifton Road, Atlanta, GA, 30333, USA; 2 Oswaldo Cruz Foundation, Rua Waldemar Falcão 121, Candeal, Salvador Bahia, 40296-710, Brazil; 3 Federal University of Bahia, Salvador, Bahia, Brazil; 4 Weill Medical College of Cornell University, 1300 York Avenue, New York, NY, 10065, USA; 5 Present address: 550 Peachtree Street NE, MOT 7, Atlanta, GA, 30308, USA

**Keywords:** Bacterial meningitis, Diagnostic test evaluation, Real-time PCR, *Streptococcus pneumoniae*, *Neisseria meningitidis*, *Haemophilus influenzae*

## Abstract

**Background:**

Although cerebrospinal fluid (CSF) culture is the diagnostic reference standard for bacterial meningitis, its sensitivity is limited, particularly when antibiotics were previously administered. CSF Gram staining and real-time PCR are theoretically less affected by antibiotics; however, it is difficult to evaluate these tests with an imperfect reference standard.

**Methods and findings:**

CSF from patients with suspected meningitis from Salvador, Brazil were tested with culture, Gram stain, and real-time PCR using *S. pneumoniae, N. meningitidis,* and *H. influenzae* specific primers and probes. An antibiotic detection disk bioassay was used to test for the presence of antibiotic activity in CSF. The diagnostic accuracy of tests were evaluated using multiple methods, including direct evaluation of Gram stain and real-time PCR against CSF culture, evaluation of real-time PCR against a composite reference standard, and latent class analysis modeling to evaluate all three tests simultaneously.

**Results:**

Among 451 CSF specimens, 80 (17.7%) had culture isolation of one of the three pathogens (40 *S. pneumoniae,* 36 *N. meningitidis,* and 4 *H. influenzae*), and 113 (25.1%) were real-time PCR positive (51 *S. pneumoniae,* 57 *N. meningitidis,* and 5 *H. influenzae*). Compared to culture, real-time PCR sensitivity and specificity were 95.0% and 90.0%, respectively. In a latent class analysis model, the sensitivity and specificity estimates were: culture, 81.3% and 99.7%; Gram stain, 98.2% and 98.7%; and real-time PCR, 95.7% and 94.3%, respectively. Gram stain and real-time PCR sensitivity did not change significantly when there was antibiotic activity in the CSF.

**Conclusion:**

Real-time PCR and Gram stain were highly accurate in diagnosing meningitis caused by *S. pneumoniae, N. meningitidis,* and *H. influenzae,* though there were few cases of *H. influenzae*. Furthermore, real-time PCR and Gram staining were less affected by antibiotic presence and might be useful when antibiotics were previously administered. Gram staining, which is inexpensive and commonly available, should be encouraged in all clinical settings.

## Background

Despite advances in management, bacterial meningitis remains a severe infection with high rates of morbidity and mortality [1]. Early clinical suspicion and implementation of appropriate antimicrobial therapy are critical to minimize adverse outcomes. Cerebrospinal fluid (CSF) culture is considered the diagnostic reference standard for bacterial meningitis, and bacterial isolation is important for antimicrobial susceptibility testing and molecular epidemiology. However, CSF culture requires at least a day or more, and has limited sensitivity. Reported CSF culture sensitivities typically range between 70% to 90% [2-4], with variation in case inclusion criteria, patient characteristics, laboratory practices, and spectrum of bacterial pathogens likely contributing to the observed differences. Administration of antibiotics prior to lumbar puncture is a common situation leading to decreased culture yield [1,2,5-7]. In practice, lumbar puncture can be delayed, resulting in antibiotics given prior to CSF collection. Administration of oral antibiotics prior to admission might also occur if the patient previously sought outpatient treatment or if antibiotics are available without a physician prescription.

Gram staining, a mainstay of bacterial meningitis diagnosis, is widely available, inexpensive and rapid [1]. Reported sensitivities of CSF Gram staining range from 60% to 90% [1,6-9], although it can difficult to assess its actual performance with an imperfect reference standard. Real-time polymerase chain reaction (RT-PCR) of CSF has been suggested as a rapid diagnostic test for bacterial meningitis [1,10-12], and amplification of DNA from non-viable bacteria could potentially facilitate diagnosis in culture negative cases [12].

We evaluated the clinical accuracy of RT-PCR and Gram staining for the diagnosis of *Streptococcus pneumoniae, Neisseria meningitidis,* and *Haemophilus influenzae* meningitis in patients in Salvador, Brazil. An antibiotic detection disk bioassay was also performed on CSF to detect the presence of antibiotic activity. Since the imperfect sensitivity of CSF culture presents a challenge when using it to evaluate tests that might be more sensitive, sensitivity and specificity estimates from multiple methods were compared. RT-PCR and Gram stain were compared to a culture reference standard, and RT-PCR was further evaluated using a composite reference standard (CRS) of culture and Gram stain. A CRS addresses an imperfect reference standard by adding a second test [13]. Latent class analysis (LCA) models were used to evaluate all three tests. LCA modeling can assess multiple diagnostic tests simultaneously and has been used to evaluate tests for other infections with imperfect reference standards [13,14].

## Methods

### Ethics statement

This protocol was approved by the institutional review boards of FIOCRUZ and Weill Medical College of Cornell University, and the protocol was exempt from requiring institutional review board approval at the US Centers for Disease Control and Prevention (CDC) because it was determined to be non-human research for CDC collaborators. Written informed consent was obtained from all participants of this study, except in the situation where the participant was unable to give written informed consent due to illness. When the participant was unable give consent due to illness, written informed consent was obtained from the subject’s legally authorized representative.

### Study population and routine clinical testing

According to state health department guidelines, suspected cases of meningitis in the metropolitan region of Salvador, a city of over 2 million inhabitants in northeast Brazil, are evaluated at the emergency department of a single public infectious diseases hospital (Hospital Couto Maia, Salvador, Bahia) [15]. As per routine hospital clinical guidelines, lumbar puncture was performed on all patients with suspected acute meningitis, defined as fever > 38°C of duration < 21 days and physical exam findings of either meningismus or altered mental status. CSF specimens were tested according to laboratory guidelines to the extent possible with available specimen amount. Routine testing of CSF included leukocyte, glucose, and protein counts. Gram staining was indicated for all CSF specimens with elevated leukocyte counts (> 5 × 10^6^ cells/L, or > 15 × 10^6^ cells/L for infants aged < 30 days old), polymorphonuclear cell (PMN) predominance (> 50% of leukocytes), or abnormal chemistry results (glucose < 2.2 mmol/L, or protein > 0.65 g/L). Culture was indicated for CSF specimens with a leukocyte count > 1000 × 10^6^ cells/L, PMN predominance, or abnormal chemistry results as defined above. Gram staining and cultures were also performed outside of routine guidelines when requested by the clinician or clinical microbiologist. Latex agglutination testing for bacterial antigens was variably performed on CSF specimens depending on test kit availability (various commercial kits used) and clinician request.

As a part of an active, hospital-based surveillance program for acute meningitis established in Salvador [15], surveillance staff reviewed daily laboratory records to identify patients meeting one of the following surveillance criteria: total CSF leukocyte count > 100 × 10^6^ cells/L or abnormal CSF chemistry as defined above. A standardized case report form was used to collect clinical and epidemiologic data from medical records and when possible, patient or family interview. If the volume of CSF collected was more than adequate for routine testing, 0.5-1 mL was saved for testing with RT-PCR and the antimicrobial detection assay at the Oswaldo Cruz Foundation (FIOCRUZ, Salvador, Brazil).

Patients meeting surveillance criteria from April 10, 2006 through December 31, 2008 were considered for this analysis. Patients with specimens without results for any of the three tests under evaluation (i.e., missing results or not tested with CSF Gram stain, culture or RT-PCR for any reason) were excluded. Patients not admitted to the emergency department or inpatient ward of Couto Maia Hospital were also excluded (i.e., CSF specimens obtained from patients evaluated elsewhere but submitted to the Couto Maia Hospital laboratory).

### Real-time PCR

DNA was extracted from CSF with the QIAGEN DNA Mini kit (QIAGEN Inc., Valencia, CA). To facilitate full lysis of gram-positive bacteria, a modified protocol was used. Briefly, 200 μl of CSF was added to 100 μl of Tris-EDTA buffer containing 0.04 g/ml lysozyme and 75 U/ml of mutanolysin (Sigma, St. Louis, MO), and the mixture was incubated for 1 hour in a 37°C water bath. All subsequent steps were performed according to the QIAGEN DNA Mini kit manufacturer protocol [16]. DNA was eluted in 100 μl of QIAGEN elution buffer and stored at −20°C.

The *lytA* gene of *S. pneumoniae*[16], *ctrA* gene of *N. meningitidis*[17], and *bexA* gene of *H. influenzae*[18] were used as species-specific targets, and a positive result was indicated by amplification with an exponential increase in fluorescence in separate reactions. The primer and probe sequences are listed in Table 1. The assays were carried out in a 25 μl reaction volume with 2 μl of sample DNA and were performed by use of the TaqMan Universal Master Mix kit (Applied Biosystems, Foster City, CA), according to manufacturer instructions. No-template controls, *S. pneumoniae, N. meningitidis*, and *H. influenzae*-positive controls, and extracted water negative controls were included in every run. DNA was amplified with the 7500 Real Time PCR system (Applied Biosystems) by using the following temperature program: 50°C for 2 min, 95°C for 10 min, followed by 50 cycles of 95°C for 15 s and 60°C for 1 min. Amplification data were analyzed by instrument software (Applied Biosystems). Positive specimens were defined as those with a cycle threshold (CT) value of <36. Negative specimens were defined as those with CT values >40. Specimens with CT values from 36–40 were deemed equivocal and rerun at 1:4 dilution to dilute any PCR inhibitors that might be present. The rerun CT value was interpreted with the same cutoffs; specimens with repeat CT values still in the equivocal range were considered indeterminate. If a specimen was positive for more than one gene target, RT-PCR was repeated to confirm the results. If only one target was positive on repeat testing, the specimen was considered positive for that target only. Staff conducting the RT-PCR assays were blinded to the results of routine laboratory testing and other patient clinical data.

**Table 1:** **Real-time PCR primers and probes**[16-18]

**Oligonuclotide**	**Sequence**	**Final conc. (nM)**
*ctrA forward*	5′-TGTGTTCCGCTATACGCCATT-3′	300
*ctrA reverse*	5′-GCCATATTCACACGATATACC-3′	900
*ctrA probe*	5′-FAM-AACCTTGAGCAA″T″CCATTTATCCTGACGTTCT-3′-SpC6*	100
*bexA forward*	5′-TGCGGTAGTGTTAGAAAATGGTATTATG-3′	600
*bexA reverse*	5′-GGACAAACATCACAAGCGGTTA-3′	600
*bexA probe*	5′-FAM-ACAAAGCGTATCAA″T″ACTACAACGAGACGCAAAAA-3′-SpC6*	100
*lytA forward*	5′-ACGCAATCTAGCAGATGAAGCA-3′	200
*lytA reverse*	5′-TCGTGCGTTTTAATTCCAGCT	200
*lytA probe*	5′-FAM-TGCCGAAAACGCTTGATACAGGGAG-3′-BHQ1	200

* The quencher BHQ-1 is attached to the internal thymine residue “T”.

### Antibiotic detection

An antibiotic detection bioassay was performed to detect antibiotic presence in CSF specimens. Briefly, an inoculum of a pure culture of a pan-sensitive *Micrococcus luteus* strain (ATCC 7468) was calibrated to a McFarland standard of 0.5. A cotton-tipped sterile applicator was used to inoculate a nutrient agar plate for confluent growth. The inoculum was allowed to dry for 5 minutes before placing two sterile 6-mm filter paper disks on the plate approximately 3 cm apart. 20 μl of CSF was placed on one disk and 20 μl of sterile saline was placed on the other. Plates were incubated at 37°C in air atmosphere for 18–24 hours and examined for a zone of inhibition. A positive result was defined as the presence of any inhibition zone around the CSF-inoculated disk.

### Data analyses

For comparison of patients tested with RT-PCR with those that were not tested, categorical variables were compared using the chi-square or Fisher’s exact test. Continuous variables were compared using the Wilcoxon-Mann–Whitney test. Tests of significance were 2-tailed and a *p* ≤0.05 was considered to be significant.

For sensitivity and specificity analyses, a positive test was defined as a test result indicative of *S. pneumoniae, N. meningitidis,* or *H. influenzae* in CSF. For culture, a positive result was defined as the isolation of one of the three pathogens. A positive Gram stain result was defined as the report of gram-positive diplococci (suggestive of *S. pneumoniae*)*,* gram-negative diplococci (suggestive of *N. meningitidis*)*,* or gram-negative pleomorphic coccobacilli (suggestive of *H. influenzae*). All other culture and Gram stain results, including the absence of bacteria or the presence of other bacterial species (in the absence of *S. pneumoniae, N. meningitidis,* and *H. influenzae),* were considered a negative test result. Positive RT-PCR assays were defined as those positive for one of the three pathogens as described above. Specimens with indeterminate RT-PCR results were considered negative in all analyses. Species-specific estimates of test accuracy were also performed with positive test results defined as those indicative of the specific species being considered, and a negative result was defined as the absence of any result indicative of that particular species (i.e., no bacteria present or presence of other bacterial species in the absence of the species being considered).

Sensitivity and specificity of RT-PCR and Gram staining were calculated using classical 2 × 2 validation analysis using a CSF culture reference standard. RT-PCR performance was also evaluated using 2 × 2 validation with a CRS of CSF culture and Gram stain. For each patient, a positive CRS required the presence of a positive culture or Gram stain result. A negative CRS required negative culture and Gram stain results as defined above. A 95% exact binomial confidence interval was calculated for each parameter derived from classical validation and CRS analyses.

Latent class analyses (LCA) was used to estimate the clinical sensitivity and specificity of culture, Gram stain, and RT-PCR simultaneously. LCA fits a model to data from multiple diagnostic tests to model the prevalence of a latent class (disease status) in the analyzed population. Sensitivity and specificity estimates are based on conditional response probabilities predicted by the model [14]. For the primary LCA model, the latent class was the presence or absence of meningitis caused by *S. pneumoniae, N. meningitidis,* or *H. influenzae.* For each model, a bootstrap *p* value greater than 0.1 was considered indicative of a good fit with the observed data. Correlation between diagnostic tests not explained by the model were considered absent if the bivariate residual between test pairs was <3.84. Standard errors (SE) for each test parameter and disease prevalence estimate were provided by the LCA model and were used to calculate 95% confidence intervals (estimate +/− 1.96*SE). LCA was performed using Latent Gold 4.0 (Statistical Innovations; Belmont, MA) and all other analyses were performed using SAS 9.2 (SAS Institute Inc.; Cary, NC).

## Results

### Study population

**Figure 1:**
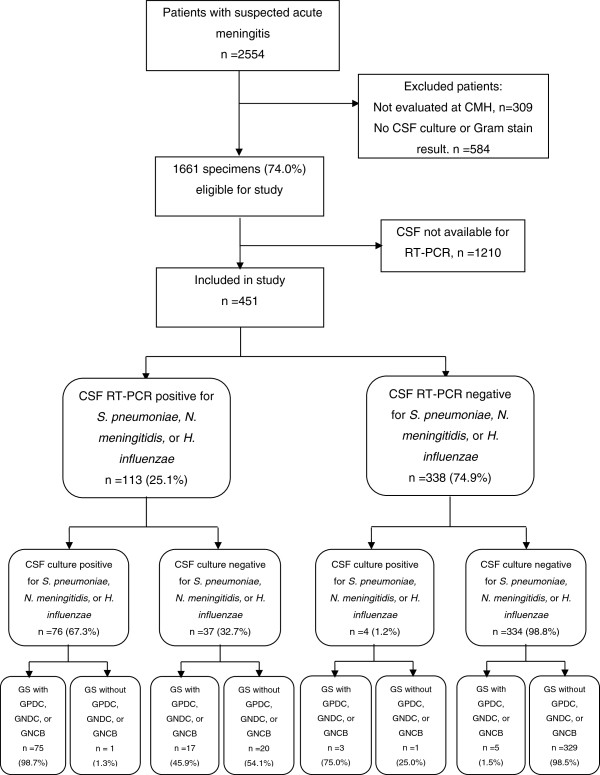
**Participant inclusion, RT-PCR, and culture results flow diagram.** Following exclusion of patients not meeting inclusion criteria and specimens that were unavailable for RT-PCR testing, the RT-PCR and culture results were analyzed for 451 specimens. Abbreviations: CMH, Couto Maia Hospital; CSF, cerebrospinal fluid; RT-PCR, real-time polymerase chain reaction; GS, Gram stain; GPDC, gram-positive diplococci; GNDC, gram-negative diplococci; GNCB, gram-negative pleomorphic coccobacilli.

From April 10, 2006 through December 31, 2008, a total of 2,554 patients met surveillance criteria, and 1,661 of these patients were evaluated at Couto Maia Hospital and had complete CSF Gram stain and culture results. Among these study eligible patients, RT-PCR was done on 451 (27.2%) of their specimens (Figure 1). Comparison of patients with CSF tested with RT-PCR with those not tested with RT-PCR showed that patients whose CSF was tested with RT-PCR were more likely to be transferred from another hospital, have an altered mental status, have higher CSF leukocyte counts, have lower CSF glucose levels, have bacteria on Gram stain, and have an isolate from CSF culture (Table 2).

**Table 2:** Demographic, clinical, and laboratory characteristics of patients included in this study

**Demographic and clinical characteristics**	**Analyzed cases (n =451)**	**Cases without RT-PCR (n=1210)**	
Male (%)	284 (63.0)	731 (60.8)	
Median age in years (range)	11 (0–78)	11 (0–83)	
Days with symptoms before admission (median, range)	2 (0–60)	2 (0–120)	
Admitted to intensive care unit (%)	56 (13.4)	104 (10.3)	
Altered mental status on admission (%)	133 (33.1)	262 (25.9)	*p* =0.007
Died (%)	42 (10.2)	95 (9.5)	
Transferred from another hospital (%)	270 (60.8)	634 (52.6)	*p* =0.003
**CSF cell counts and chemistry**			
Leukocyte count (%)			*p* =0.001
<10 x10^6^ cells/L	2 (0.4)	7 (0.6)	
10-99 x10^6^ cells/L	4 (0.9)	17 (1.5)	
100-999 x10^6^ cells/L	260 (57.8)	743 (64.3)	
1000-9,999 x10^6^ cells/L	107 (23.8)	262 (22.7)	
≥10,000 x10^6^ cells/L	77 (17.1)	127 (11.0)	
>50% PMN (%)	302 (67.1)	725 (62.7)	
>1.00 g/L protein (%)	242 (53.7)	621 (51.5)	
≤1.9 mmol/L glucose (%)	136 (30.2)	303 (25.1)	*p* =0.04
**Gram stain results**			
Gram stain with any bacteria (%)	116 (25.7)	208 (17.2)	*p* <0.0001
GPDC, GNDC, or GNCB (%)	100 (22.2)	172 (14.2)	
GPDC (%)	49 (10.9)	80 (6.6)	
GNDC (%)	46 (10.2)	80 (6.6)	
GNCB (%)	5 (1.1)	12 (1.0)	
Gram stain with bacteria of other morphology	16 (3.6)	36 (3.0)	
**Culture results**			
Positive for any bacteria (%)	100 (22.2)	178 (14.7)	*p =*0.0003
*S. pneumoniae, N. meningitidis,* or *H. influenzae* (%)	80 (17.7)	143 (11.8)	
*S. pneumoniae* (%)	40 (8.9)	61 (5.0)	
*N. meningitidis* (%)	36 (8.0)	67 (5.5)	
*H. influenzae* (%)	4 (0.9)	15 (1.2)	
*Streptococcus agalactiae* (%)	2 (0.4)	2 (0.2)	
Other *Streptococcus* species (%)	1 (0.2)	4 (0.3)	
*Staphylococcus* species (%)	5 (1.1)	4 (0.3)	
*Escherichia coli* (%)	2 (0.4)	5 (0.4)	
*Listeria monocytogenes* (%)	1 (0.2)	0	
*Salmonella* species (%)	1 (0.2)	0	
Other bacteria	8 (1.8)	20 (1.7)	
**RT-PCR result**			
Any positive (%)	113 (25.1)	-	
*S. pneumoniae* (%)	51 (11.3)	-	
*N. meningitidis* (%)	57 (12.6)	-	
*H. influenzae* (%)	5 (1.1)	-	

Patients included in this study are compared to those who were excluded due to the absence of CSF RT-PCR testing.

Note. Denominators may vary due to incomplete data.

Abbreviations: RT-PCR, real-time polymerase chain reaction; CSF, cerebrospinal fluid; PMN, polymorphonuclear leukocyte; GPDC, gram-positive diplococci; GNDC, gram-negative diplococcic; GNCB, gram-negative pleomorphic coccobacilli.

## Test results

Among 451 patients with CSF Gram stain, culture, and RT-PCR done, 80 (17.7%) had CSF culture isolation of *S. pneumoniae, N. meningitidis,* or *H. influenzae* (Tables 2 and 3)*.* One hundred patients (22.2%) had a CSF Gram stain suggestive of *S. pneumoniae, N. meningitidis,* or *H*. *influenzae.* RT-PCR of CSF was positive for 113 (25.1%) patients, of which 37 (32.7%) had negative culture results (as per study definition), 39 (34.5%) had *S. pneumoniae* isolated, 34 (30.0%) had *N. meningitidis* isolated, and 3 (2.7%) had *H. influenzae* isolated (Figure 1). Four specimens had indeterminate RT-PCR results (two for *lyt*A and two for *ctrA*), and these results were considered negative in all analyses. RT-PCR was negative in all 20 CSF specimens that with culture isolation of bacteria other than *S. pneumoniae, N. meningitidis,* or *H. influenzae.* Among 96 specimens with a positive result for two or more of the diagnostic tests being considered*,* only one had positive results suggestive of different species when interpreted with study definitions. This specimen had a sterile culture, positive RT-PCR for *N. meningitidis,* and a Gram stain read as having gram-positive diplococci.

**Table 3:** Test result patterns for CSF culture, Gram stain, and RT-PCR for analyzed patients (total n=451)

**Culture**	**Gram stain**	**RT-PCR**	**n**
-	-	-	329
-	-	+	20
-	+	-	5
-	+	+	17
+	-	-	1
+	-	+	1
+	+	-	3
+	+	+	75

Note. Positive test results (+) for culture or RT-PCR defined as identification of *N. meningitidis, S. pneumoniae,* or *H. influenzae.* Positive results (+) for Gram stains defined as report of bacteria with characteristic morphology and staining suggestive of *N. meningitidis, S. pneumoniae,* or *H. influenzae.* Any result not meeting the positive result definitions are denoted as negative (−).

### Sensitivity and specificity estimates

Sensitivity and specificity estimates are summarized in Table 4. Using a culture reference standard, sensitivity and specificity of RT-PCR were 95.0% (95% CI 87.7-98.6%) and 90.0% (95% CI 86.5-92.9%), and Gram stain sensitivity and specificity were 97.5% (95% CI 91.3-99.7%) and 94.1% (95% CI 91.2-96.3%), respectively. RT-PCR sensitivity decreased and specificity increased to 94.3% (95% CI 91.3-96.5%) when compared against the CRS. In a LCA model with CSF culture, Gram stain, and RT-PCR (Table 4), the modeled prevalence of meningitis caused by *S. pneumoniae, N. meningitidis,* or *H. influenzae* was 21.5% (95% CI 17.7-25.4), and culture sensitivity (81.3%; 95CI 73.2-89.3) was less than that of Gram stain (98.2%; 95% CI 95.2-100.0%) and RT-PCR (95.7%; 95% CI 91.2-100.0%). RT-PCR specificity in the LCA model was 94.3% (95% CI 91.9-96.8%).

**Table 4:** **Sensitivity and specificity estimates for diagnosis of *S. pneumoniae, N. meningitidis, *or *H. influenzae *meningitis**

			**Culture Standard**		**CRS**^**a**^		**LCA Model**^**b**^	
			**Sens (%, 95CI)**	**Spec (%, 95CI)**	**Sens (%, 95CI)**	**Spec (%, 95CI)**	**Sens (%, 95CI)**	**Spec (%, 95CI)**
***S. pneumoniae, N. meningitidis,*or*H. influenzae***								
All patients, n=451		**Culture**	na	na	na	na	81.3 (73.2-89.3)	99.7 (99.1-100.0)
		**Gram stain**	97.5 (91.3-99.7)	94.1 (91.2-96.3)	na	na	98.2 (95.2-100.0)	98.7 (97.3-100.0)
		**RT-PCR**	95.0 (87.7-98.6)	90.0 (86.5-92.9)	91.2 (83.9-95.9)	94.3 (91.3-96.5)	95.7 (91.2-100.0)	94.3 (91.9-96.8)
Antibiotic negative, n=341		**Culture**	na	na	na	na	85.7 (74.4-96.9)	100.0 (99.8-100.0)
		**Gram stain**	100.0 (89.7-100.0)	97.4 (94.9-98.9)	na	na	98.7 (94.9-100.0)	99.0 (97.9-100.0)
		**RT-PCR**	97.1 (84.7-99.9)	96.1 (93.3-98.0)	90.5 (77.4-97.3)	97.7 (95.2-99.1)	95.8 (89.1-100.0)	97.7 (95.9-99.4)
Antibiotic positive, n=98		**Culture**	na	na	na	na	78.2 (66.4-89.9)	97.1 (91.9-100.0)
		**Gram stain**	97.7 (88.0-99.9)	74.1 (60.4-85.0)	na	na	99.5 (97.3-100.0)	94.0 (84.1-100.0)
		**RT-PCR**	93.2 (81.3-98.6)	55.6 (41.4-69.1)	91.4 (81.0-97.1)	70.0 (53.5-83.4)	95.3 (89.0-100.0)	70.2 (56.2-84.2)
***S. pneumoniae*only**								
All patients, n=451		**Culture**	na	na	na	na	85.3 (74.8-95.9)	100.0 (99.9-100.0)
		**Gram stain**	97.5 (86.8-99.9)	97.6 (95.6-98.8)	na	na	96.4 (90.6-100.0)	99.0 (98.1-100.0)
		**RT-PCR**	97.5 (86.8-99.9)	97.1 (95.0-98.5)	90.0 (78.2-96.7)	98.5 (96.8-99.5)	96.4 (90.6-100.0)	98.6 (97.4-99.7)
***N. meningitidis*only**								
All patients, n=451		**Culture**	na	na	na	na	77.9 (65.4-90.4)	99.8 (99.3-100.0)
		**Gram stain**	97.2 (85.5-99.9)	97.4 (95.3-98.7)	na	na	98.7 (94.8-100.0)	99.6 (98.9-100.0)
		**RT-PCR**	94.4 (81.3-99.3)	94.5 (91.8-96.5)	91.5 (79.6-97.6)	96.5 (94.3-98.1)	95.9 (89.3-100.0)	96.6 (94.8-98.3)

Performance parameters for the various tests are estimated using a CSF culture reference standard, composite reference standard (CRS), and latent class analysis (LCA) modeling with stratification by antimicrobial detection disk bioassay results.

Abbreviations: CRS, composite reference standard; LCA, latent class analysis; na, not applicable; 95% CI, 95% exact binomial confidence interval for culture reference standard and CRS parameters, or parameter estimate +/− 1.96 * standard error for LCA models.

^a^Composite reference standard defined as positive if CSF culture or Gram stain positive for *S. pneumoniae, N. meningitidis,* or *H. influenzae.*

^b^For LCA model of all patients, disease prevalence was 21.5% (95% CI 17.7-25.4). For the model of antibiotic assay-negative patients, disease prevalence was 11.6% (95CI 8.2-15.0). For the model of antibiotic assay-positive patients, disease prevalence was 55.8% (96CI 45.4-66.1). For the model of *S. pneumoniae* diagnosis, disease prevelance was 10.4% (95CI 7.5-13.2). For the model of *N. meningitidis* diagnosis, disease prevalence was 10.0% (95CI 7.2-12.7). Bootstrap *p* for all models >0.1.

Among 439 specimens tested with the antimicrobial detection disk bioassay, 98 (22.3%) tested positive. Sensitivity and specificity estimates following stratification of patients based on the bioassay result are shown in Table 4. There was minimal decrease in Gram stain and RT-PCR sensitivity in specimens with evidence of antimicrobial activity, while there was a large decrease in RT-PCR specificity among specimens with evidence of antimicrobial activity. Culture sensitivity decreased among specimens with evidence of antimicrobial activity. Species-specific test parameters for *S. pneumoniae* and *N. meningitidis* were similar to those determined for all three pathogens considered together (Table 4). *H. influenzae-*specific test parameters were not calculated due to the low numbers of positive tests.

All LCA models achieved adequate fit based on bootstrap *p* values criteria, and correlations between diagnostic tests were adequately explained by the models based on bivariate residual criteria.

### Patients with isolated RT-PCR-positive CSF

Patients with CSF specimens that were positive by RT-PCR with negative cultures and Gram stains for *S. pneumoniae, N. meningitidis,* and *H*. *influenzae* (n=20) are compared with patients with all negative and all positive test results in Table 5. Test results for the patients with an isolated positive RT-PCR result were most similar with those from patients with positive results for all three diagnostic tests. Latex agglutination test results for bacterial antigens were available for 13 of the patients with an isolated positive RT-PCR result. Five of these latex agglutination tests were positive, and all positive results were concordant with the pathogen identified by RT-PCR.

**Table 5:** Characteristics of patients with isolated positive RT-PCR results

	**Culture-Gram-PCR result**^**b**^		
	**All tests negative**	**Isolated RT-PCR positive**	**All tests positive**
	**(n=329)**	**(n=20)**	**(n=75)**
Age in years (median, range)	9 (0–64)	11 (0–63)	15 (0–78)
Altered mental status (%)	66 (22.6)	6 (40.0)	47 (66.2)
Deaths (%)	27 (9.1)	2 (10.5)	10 (14.3)
Median WBC (x10^6^ cells/L, range)^c^	430 (100–10,000)	10,000 (32–10,000)	8,600 (0–10,000)
>50% PMN cells (%)	186 (56.7)	18 (90.0)	72 (96.0)
Median protein (g/L, range)^d^	0.50 (0.20-5.00)	2.90 (0.32-5.00)	3.50 (0.28-5.00)
Median glucose (mmol/L, range)	2.9 (1.1-3.9)	1.6 (0.6-3.1)	1.1 (1.1-3.2)
LAT positive (% of total LAT performed)	1 (2.0)	5 (38.5)	25 (80.6)
Positive LAT concordant with RT-PCR (%)^e^	n/a	5 (100.0)	25 (100.0)
Antibiotic bioassay positive (%)	28 (8.8)	12 (63.2)	41 (55.4)

Patients with isolated positive RT-PCR results are compared with patients with all negative or all positive culture, Gram stain, and RT-PCR test results.^a^

Abbreviations: WBC, white blood cells; PMN, polymorphonuclear; LAT, latex agglutination test; n/a, not applicable.

^a^Patients with missing data exclude from calculations.

^b^Positive or negative for *N. meningitidis, S. pneumoniae,* or *H. influenzae* as defined in Methods*.*

^c^Maximum value truncated at 10,000 x10^6^ cells/L.

^d^Maximum value truncated at 5.00 g/L.

^e^Number and percentage of positive latex agglutination test results that were consistent with RT-PCR result.

## Discussion

RT-PCR and Gram staining were highly accurate for *S. pneumoniae, N. meningitidis,* and *H*. *influenzae* meningitis diagnosis with sensitivity and specificity estimates above 90% in all analytic approaches. Although CSF culture is considered the diagnostic reference standard for bacterial meningitis, its limited sensitivity often results in an inability to confirm the diagnosis and target antimicrobial therapy. The imperfect sensitivity of culture also presents a difficulty when evaluating other tests that are potentially more sensitive. Composite reference standards and LCA modeling, which can estimate test performance parameters of multiple tests simultaneously, can be helpful in addressing this problem. We are unaware of previous published studies what have used LCA modeling to evaluate the clinical accuracy of tests for acute bacterial meningitis, an approach that requires no assumptions about the accuracy of CSF culture and furthermore, allows us to also compare the sensitivity of CSF culture with the comparison tests. The sensitivities of RT-PCR and Gram staining were over 95% and far exceeded the 81% sensitivity of culture when estimated using LCA models. LCA models stratified by results of the antibiotic detection bioassay also provide evidence that RT-PCR and Gram stain sensitivity are less affected by the presence of antibiotic activity in the CSF. This is consistent with a recent study that found the presence of antibiotic activity in CSF is a strong risk factor for a RT-PCR positive, culture-negative case [12]. These findings suggest that RT-PCR might be particularly useful in settings where patients often receive antibiotics prior to lumbar puncture.

Nucleic acid amplification tests such as PCR do not require viable bacteria for a positive assay and are generally considered to be highly sensitive [14]. Nevertheless, the presence of PCR inhibitors in clinical specimens can compromise sensitivity [19], and the presence of inhibitors might explain the imperfect RT-PCR sensitivity in this and other studies [1]. When compared to a culture reference standard, RT-PCR specificity in this study was 90%. While contamination of RT-PCR reactions leading to false-positive results is always a concern, stratification of subjects by the presence or absence of antibiotic activity in the CSF resulted in RT-PCR specificities of 56% and 96%, respectively. This suggests that the observed RT-PCR specificity is negatively biased by the imperfect sensitivity of culture, particularly when antibiotics have been administered. RT-PCR specificities calculated using a CRS or LCA modeling were higher, suggesting that these approaches can help address the bias introduced by the insensitivity of culture. Nonetheless, it appears that RT-PCR specificity is still significantly underestimated by these alternative analytic approaches. The LCA model assigned a high probability (>98%) of a disease-free status to patients with specimens testing positive with RT-PCR only. However several of these patients had additional evidence supporting a diagnosis of bacterial meningitis (i.e. positive latex agglutination tests and elevated CSF leukocyte counts). This apparent misclassification would result in falsely low RT-PCR specificity and overestimated culture and Gram stain sensitivities. This limitation could possibly be addressed with other CRS or LCA models that incorporate additional tests that are likely less affected by antibiotic administration, such as assays for bacterial antigens (i.e. latex agglutination tests and the rapid immunochromatographic test for *S. pneumoniae*) [20] or CSF indices (i.e. leukocyte, protein, and glucose counts).

Numerous PCR assays have been evaluated for bacterial meningitis diagnosis using species-specific primers [10-12,16,17,21-24] or broad range bacterial PCR [25,26]. While PCR-based assays have played an increasingly important role in meningitis surveillance in Brazil [12] and elsewhere, including the United Kingdom [27], a routine role in clinical practice has not been established. This is likely due to the resources required and empiric treatment regimens that make confirmation less critical. Current automated RT-PCR systems have advantages over traditional PCR that make it attractive in the clinical setting, including rapid availability of results within hours and a closed system that can reduce contamination risk [28]. Although conventional antimicrobial susceptibility testing requires that the pathogen be cultured, RT-PCR identification of bacterial pathogens might allow some narrowing of antibiotic treatment in clinical situations where cultures are negative. Accuracy of RT-PCR testing could be further improved by using newer RT-PCR primers with improved sensitivity [24] or performing secondary testing with other RT-PCR assays when there is an indeterminate result. However, given the potentially severe consequences of misdiagnosis, the strengths and limitations of any test should be carefully considered before recommending new diagnostic strategies.

CSF Gram staining, long considered a mainstay of bacterial meningitis diagnosis [1], is rapid, inexpensive, and requires relatively little training. However, accurate results are highly dependent on the operator’s staining and interpretation skills. Previous studies report Gram stain sensitivities as high as 90% and specificities of 97% or more [8,29]. In the LCA model in this study, Gram staining had a sensitivity of 98.2% and specificity of 98.7%. As previously discussed, the likely misclassification of several patients with an isolated positive RT-PCR result as disease-free suggests that the model overestimated Gram stain and culture sensitivity, and to a lesser extent, specificity. However, as an infectious diseases referral center, Couto Maia Hospital often evaluates several patients with suspected meningitis daily, and the experience of the microbiology staff likely contributed to the excellent performance of Gram staining. Given its wide availability, rapid turnaround, low cost, and high accuracy, the value of Gram staining in the clinical workup of suspected meningitis cannot be understated.

It should be noted that only *S. pneumoniae, N. meningitidis,* and *H. influenzae* were considered in this study*.* The performance of Gram stain and CSF culture in diagnosing other bacterial meningitis pathogens might differ significantly. Furthermore, because there were few *H. influenzae* cases, due to routine Hib vaccination in infants, the test parameter estimates are likely most valid for *S. pneumoniae* and *N. meningitidis*. Also, local clinical testing guidelines may have resulted in a unique study population in this study, as CSF culture was not routinely recommended for CSF specimens not meeting specific criteria. Interestingly, patients included in our final analysis were more likely to have clinical markers of severe illness consistent with bacterial meningitis when compared to those not included due to absent RT-PCR testing. The reasons for this are unclear; however, according to surveillance staff, the most common reasons for CSF not to be tested with RT-PCR were insufficient volume, loss of the specimen when discarded by hospital staff unfamiliar with the surveillance protocol, or frequent unavailability of specimens during a prolonged aseptic meningitis outbreak in 2007, due to prioritized testing at the state public health laboratory. Since test performance can vary with different patient characteristics, evaluation of these tests in other patient populations might result in different performance parameter estimates, and the clinical characteristics of our study population should be considered when interpreting the findings of this study. However, it is important to note that test sensitivity and specificities are intrinsic parameters that would not vary with changes in disease prevalence alone.

## Conclusions

Newer technologies can often provide increasingly sensitive diagnostic tests; however, there is no straightforward method to evaluate their performance when they are more sensitive than the reference standard. Using multiple analytic approaches, we found that RT-PCR assays on CSF specimens were highly accurate for diagnosis of *S. pneumoniae, N. meningitidis,* and *H. influenzae* meningitis. RT-PCR appears to be useful in situations where antibiotics have been administered prior to lumbar puncture, and this can be particularly helpful for surveillance in settings where this is common. This study also reaffirms the value of CSF Gram staining in the evaluation of acute meningitis, and its routine use should continue to be encouraged.

## Competing interests

The authors declare that they have no competing interests.

## Authors’ contributions

HMW, BDP, TAC, SWM, and JNR analyzed the data. MGR, LWM, AIK, and JNR conceived and designed the study. JA, TQO, and MCL conducted the case surveillance and collected clinical data. SMC, BHH, MGC, KS, and JNR conducted the laboratory assays. HMW, SMC, BHH, TAC, AIK, SWM, and JNR drafted the manuscript. All authors read and approved the final manuscript.

## Pre-publication history

The pre-publication history for this paper can be accessed here:

http://www.biomedcentral.com/1471-2334/13/26/prepub
